# A System of Anatomy for the Use of Students of Medicine

**Published:** 1846-11

**Authors:** 


ART. V.—*A System of Anatomy for the Use of Students of Medicine.* By CASPAR WISTAR, M. D., late Professor of Anatomy in the University of Pennsylvania. With Notes and Additions. By WILLIAM E. HORNER, M.D., Professor of Anatomy in the University of Pennsylvania. Ninth edition. Entirely remodeled and illustrated by more than two hundred engravin s. By J. PANCOAST, M.D., Professor of General, Descriptive and Surgical Anatomy in Jefferson Medical College of Philadelphia, Lecturer on Clinical Surgery, Fellow of the Philadelphia College of Physicians, &c., &c. In two volumes. 8vo. pp. 1160. Philadelphia: Thomas, Cowperthwaite, & Co. 1846.

In 1814, Dr. Wistar, then at the height of his fame as Professor of Anatomy in the University of Pennsylvania, published the first edition of this work. As remarked in the preface by Dr. Pancoast, "Simple in its construction, concise, but yet clear, and at the same time representing faithfully and fully, the science of anatomy as it then existed, the book was exactly in keeping with the well known character of its distinguished author." It met with a rapid sale, and a second edition was called for in 1817. A year afterwards the author died, and when the publication of a third edition was demanded Dr. Horner, the personal friend of Wistar, gave to it his superintendence, and enriched it with much additional matter which the progress of the science had brought to light. Although more than twenty years have now passed away since the work took its place among our professional literature, it continues to be regarded with the highest favor, and there is not a doubt that the present edition will pass off as rapidly as any of the preceding ones. The engravings, of which the early editions had not the advantage, place it upon an equality with the illustrated works on anatomy which the modern press has brought forth. We shall rejoice in the continued prosperity of this national work, the author of which was an ornament at once to his profession and his species—"a man whose literary and professional career was so creditable to the reputation of his country, whose philanthropy and sauvity of manners established him so firmly in the affections and confidence of all who knew him," and whose eloquence has seldom been equalled by any lecturer on anatomy in any country.

